# Impact of Hepatitis E Virus Screening in the UK Deceased Organ Donor Population

**DOI:** 10.3389/ti.2023.11673

**Published:** 2023-09-04

**Authors:** Ines Ushiro-Lumb, John Forsythe, Becky Haywood, Christie Geoghegan, Victoria Maddox, Samreen Ijaz, Derek Manas, Douglas Thorburn

**Affiliations:** ^1^ Organ and Tissue Donation and Transplantation, NHS Blood and Transplant, London, United Kingdom; ^2^ UK Health Security Agency (UKHSA), London, United Kingdom; ^3^ Microbiology Services Laboratory, NHS Blood and Transplant, London, United Kingdom; ^4^ Clinical Services, NHS Blood and Transplant, London, United Kingdom

**Keywords:** donor screening, hepatitis E virus, donor-derived infection, transplant-related infection, donor-derived transmission

## Abstract

Universal Hepatitis E Virus (HEV) screening of deceased organ donors was implemented by the UK national organ procurement organisation in October 2017. Donor testing for HEV infection is done post-transplant; detection of HEV ribonucleic acid (RNA) in donor plasma is therefore not a contra-indication for organ donation, with the result being used to inform recipient management. Immediate post-transplant detection of donor HEV viraemia triggers notification to transplant centres. Follow up of liver and kidney recipients has shown that transmission through solid organs is very efficient, particularly through liver grafts, as expected; no other organ types were transplanted in this cohort. Although donors with higher plasma viral load (VL > 10^3^ IU/mL) were invariably associated with recipient infection, transmission was also documented at lower VL levels. Knowledge of donor HEV status has led to identification of transmission of infection via solid organ grafts followed by close patient monitoring and informed clinical management decisions. The purpose of this strategy is to allow early detection of infection and recurrence and treatment to circumvent the risk of accelerated liver damage from chronic HEV infection due to undiagnosed, inadvertent donor-derived transmission of infection.

## Graphical Abstract

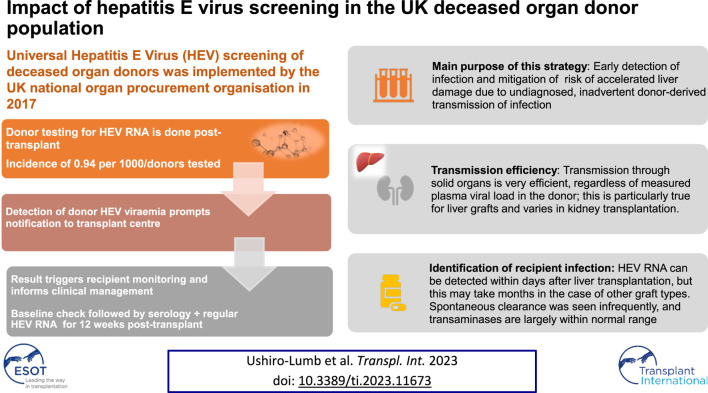

## Introduction

Hepatitis E virus (HEV) is a very common cause of acute hepatitis worldwide [1]; the epidemiology, distribution and natural history of infection differs according to the viral genotypes 1–4. Infection is asymptomatic or mild and self-limiting in most people. However, individuals with a significantly impaired immune system are at higher risk of complications, including establishment of chronic infection and accelerated progression to cirrhosis, typically caused by genotype 3 viruses. Immunocompromised individuals are at much higher risk of acquiring HEV infection from diet than from transfusion of blood components and organ transplantation, hence advice and education on control of dietary exposure remains essential. In 2016, following guidance from the UK Standard Advisory Committee on the Safety of Blood, Tissues and Organs (SaBTO), universal screening of blood donors for hepatitis E was introduced; the UK was the first country in the world to adopt this strategy and the screening of organ donors commenced in October 2017 [2]. Following the principle of a balanced approach to improve outcomes for organ transplant recipients, screening is performed post-donation.

## Patients and Methods

All potential deceased organ donors undergo mandatory infection screening at the time of donor characterisation are tested post-donation for HEV Ribonucleic Acid (RNA). This testing is done in a single reference laboratory where plasma samples are tested individually by a transcription mediated assay (TMA) according to manufacturer’s instructions (Procleix HEV assay, Grifols diagnostic solutions inc.; 95% lower limit of detection 7.89 IU/mL). Reactive samples are re-tested in an alternative molecular assay (ampliCube HEV 2.0 Quant, Mikrogen diagnostic, 95% lower limit of detection 36.13 IU/mL) and where possible, the viral load is quantified. Serology is also applied to all reactive samples (HEV-IgG Elisa, Fortress diagnostics). Transplant centres receive the screening results within an average of 5 days from the date of transplant; in addition, centres are promptly contacted in the event of positive donor results and advised to commence recipient testing, with hepatology referral. Pre-transplant recipient serum is retrospectively tested for HEV IgG to document baseline serostatus. Follow up plasma samples are taken on communication of the donor’s result, and thereafter at regular intervals when the patient is reviewed in clinic for no less than 12 weeks. These are tested for HEV RNA and IgG and the HEV infection status of each recipient is recorded centrally.

### Ethical Approval

NHSBT is reliant on the General Data Protection Regulation Article 6(1)(e)—Performance of a public task. Under Article 9(2)(h), (i), and (j), NHSBT is allowed to use patient identifiable information for service evaluation and safety monitoring without the consent of patients.

## Results

9,500 deceased potential organ donors were screened between October 2017 and October 2022, with nine confirmed viraemic cases identified; this incidence of 0.94 per 1,000 is approximately four times higher than that seen in our blood donor population. One potential donor who retrospectively tested positive for HEV RNA did not donate tissues or organs. The remaining eight proceeding donors, with plasma viral load (VL) ranging from 100 to 270,000 IU/mL, donated fourteen kidneys and six livers to twenty recipients (Table 1). All liver recipients had demonstrable HEV RNA in plasma, detected at various time points post-transplantation. which was commenced at different time points after diagnosis of HEV infection. Time of commencement, duration of treatment, ribavirin dose and dose adjustments, as well as changes in immunosuppression were determined by the teams caring for individual patients.

**TABLE 1:** Donor and recipient demographics, with outcomes of donation from HEV viraemic deceased organ donors.

Donor characteristics						Recipient characteristcs							
Donor	Age (years)	Gender	Cause of death	Donor type	HEV plasma load (IU/mL)	Recipient	Gender	Age (years)	Organ type	Pre-transplant HEV IgG	Transplant-related HEV infection	Post-transplant HEV RNA detection (days)^a^	Post ribavirin SVR
1	60	M	ICH	DCD	100	1A	F	64	Liver	Negative	Yes	11	Yes
						1B	M	62	R Kidney	Negative	Yes	74	Spontaneous clearance
						1C	M	61	L Kidney	Negative	Yes	42–106	Yes
2	44	M	ICH	DBD	3,653	2A	M	60	Liver	Negative	Transient positivity	9	n/a
						2B	M	35	R Kidney	Negative	Yes	54	Yes
						2C	M	62	L Kidney	Negative	Yes	70	Yes
3	36	M	HBD	DBD	435	3A	M	53	Liver	Negative	Yes	<10^b^	Yes
						3B	F	25	R Kidney	Negative	No	—	n/a
						3C	F	32	L Kidney	Negative	Yes	84	on ribavirin
4	60	M	HBD	DBD	287,000	4A	M	64	Liver	Negative	Yes	<10	Yes
5	57	M	HBD	DBD	98,300	5A	M	58	Liver	Negative	Yes	7	Yes
						5B	M	68	R Kidney	Positive	Yes	13–80	Yes
						5C	M	36	L Kidney	Negative	Yes	115^c^	Yes
6	58	M	HBD	DCD	436	6A	M	37	R Kidney	Negative	No	—	n/a
						6B	F	61	L Kidney	Negative	No	—	n/a
7	58	M	ICH	DBD	3,340	7A	F	38	R Kidney	Negative	Yes	<18	Yes
						7B	M	62	L Kidney	Negative	Yes	<12	RIP
8	36	M	ICH	DBD	111	8A	M	38	Liver	Positive	Probable	<10	on ribavirin
						8B	F	39	R Kidney	Negative	No	—	n/a
						8C	F	44	L Kidney	Negative	No	—	n/a

ICH, intracerebral haemorrhage; HBD, hypoxic brain damage; DCD, donation after circulatory death; DBD, donation after brain death; SVR, sustained virological response.

^a^ Time when first positive result available; does not indicate precise start of detectable viraemia in most cases. Date of last negative to first positive interval is given in some cases as first measured, viral load indicates viraemia would have been detectable between those dates.

^b^ < Viral load indicates viraemia would have been detectable before that date.

^c^ Ribavirin from day 5 to 35; regular surveillance revealed late viraemia.

**FIGURE 1:**
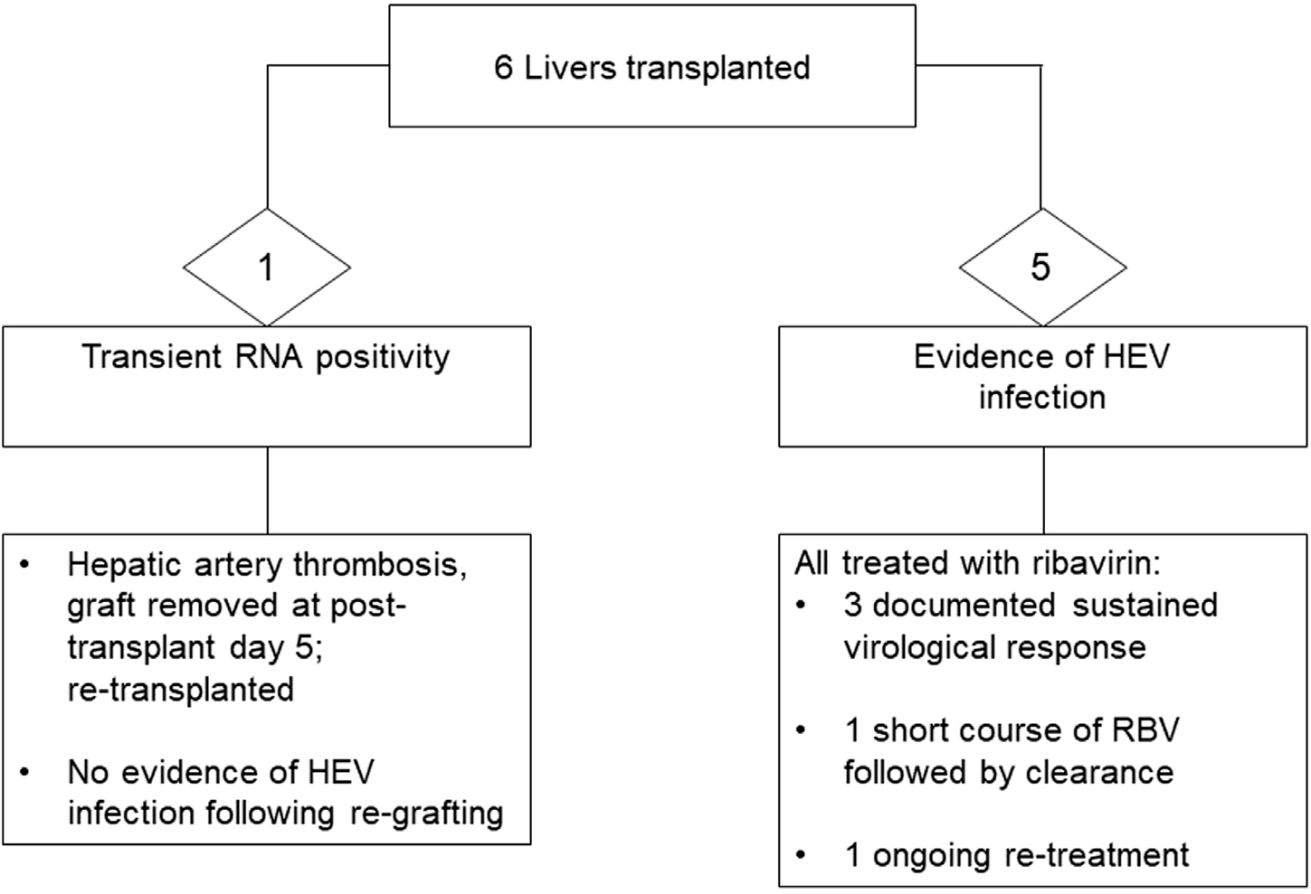
Outcome of liver donation from donors with confirmed HEV viraemia.

**FIGURE 2:**
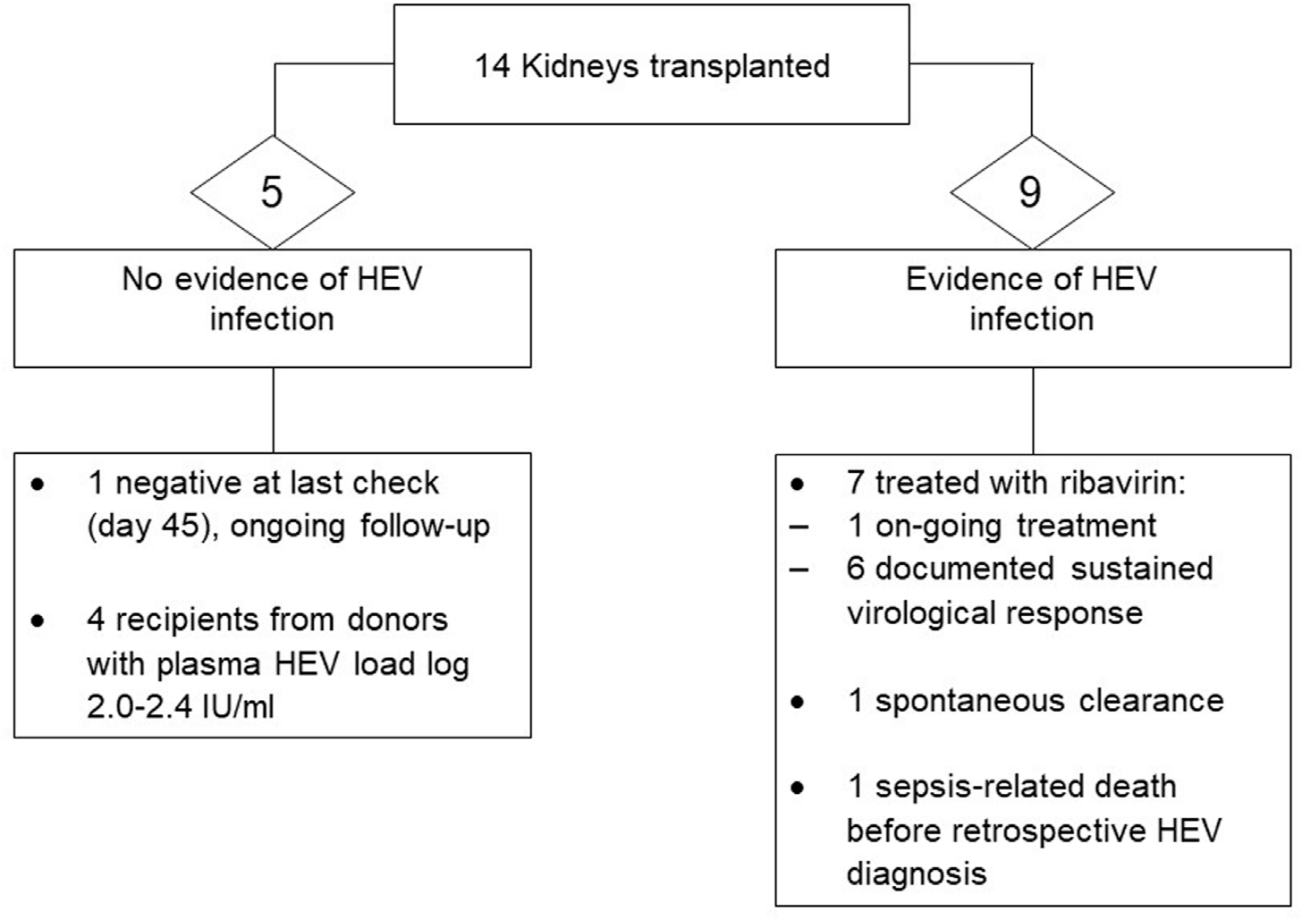
Outcome of kidney donation from donors with confirmed HEV viraemia.

Time to achieve initial negative viral RNA measurement, followed by sustained virological response (SVR, i.e., negative viral RNA in plasma and stool beyond 6 months from completion of antiviral treatment) ranged significantly, from 4 weeks up to 24 months. Rapid viral clearance (undetectable viral RNA by the locally applied standard of care methodology), with a first negative result in plasma was observed in two liver recipients who were commenced on ribavirin immediately upon detection of viraemia. Significant intolerance to ribavirin was noted in three recipients, and prolonged treatment course was required in one liver recipient who suffered from side effects requiring interruption of the drug, with virus rebound on three occasions. A snapshot of recipient outcome is shown in Figures 1, 2. Detailed recipient characteristics, their management and outcomes, as well as molecular analysis of the infecting strains, are the subject of a separate piece of work involving all the various teams and will be described elsewhere.

## Discussion

### Yield From Universal HEV RNA Screening

The introduction of universal screening was considered in the context of the relevant UK epidemiology for this zoonotic infection and its clinical impact on immunocompromised patients. The incidence of asymptomatic acute HEV infection observed in the UK blood donor population was around 1 in 2,850 donors when a large study was conducted in 2012/13 [3] with no significant changes in the immediate subsequent years and a decline from 2017, mirroring the epidemiology in the UK general population [4]. A gradual drop in incidence has been observed over more recent years, with 1 in 4,347 being the approximate figure for blood donors in 2021 [5]. Interestingly, the yield from deceased organ donor screening has been showing a different pattern, with one case of acute HEV being identified per 536, 1,682 and 1,797 donors tested in 2020, 2021, and 2022, respectively. The reason for this is not entirely clear, and not only do we continue to detect HEV viraemia in deceased donors, but we have also seen an increase in incidence during the COVID-19 pandemic. The demographics of acutely infected donors reflects the epidemiology of the general UK population, with more cases seen in men in the >50 year old age group [4]. The numbers tested are low, on average 1,600 to 1,800 potential donors per year, hence the number of identified infected donors is small, but significant; the screening strategy introduced in the UK in late 2017 has led to the identification of 20 recipients who have benefited from monitoring and tailored intervention to avoid ultimate liver damage due to late diagnosis. The approach hereby described ensured no transplant-related chronic infections were missed in the organ recipients since donor screening was initiated. Many more infections acquired through consumption of contaminated food are likely to be missed, so information and awareness amongst patients and healthcare professionals remains important.

### Thresholds of Transmission and Course of Infection in Recipients

Donor-derived HEV infection has been infrequently described, with scarce publications available in the literature [6–8]. Without organ donor screening and post-transplant recipient surveillance for HEV RNA positivity, there is a real possibility of under recognition of infection of donor origin; diagnosis of chronic HEV infection many months or years after transplantation does not necessarily trigger look back investigations. It is acknowledged however, that apart from further contribution from transfusion-associated infections, the dietary route remains the main route of acquisition of zoonotic HEV genotypes. In the setting of significant immunocompromise, absence of significant inflammatory responses with normal or mildly abnormal liver enzymes may not trigger testing, particularly in the early post-transplant period. Familiarity with local epidemiology and need to include HEV in testing panels, where appropriate, can address some of the issues with under ascertainment and late diagnosis.

There is no definition of infectious dose in the context of an infected organ being used for transplantation, and presence of viable virus within the graft is theoretically sufficient to pose a transmission risk; no data exist to suggest thresholds of transmission based on measured plasma viral load in the donor and indeed, low plasma loads in our donor cohort were associated with transmission through not only liver, as would be expected, but also kidneys (Table 1). Risk of transmission is of course multifactorial, but some observations from this cohort are worthy of mention. As viable virus will be present in the liver, viral load in plasma during early acute and early resolving infection in the donor cannot be used to stratify risk of transmission through this organ; as seen in our cases, low level VL in the order of 10^2 IU/mL resulted in transmission through the liver but not through kidneys from the same donor. Transmission via an infected liver graft with undetectable viral RNA in plasma has been described [6]. Determinants of transmission and control of infection have not been defined but both viral and host factors are expected to play a role; this includes the net immune status of recipients as regards to control of viral infections. Previously described recipient characteristics that are linked to progression to HEV chronicity include lower lymphocyte count and exposure to tacrolimus [9]; detailed variables are also being collated for this cohort.

Where local epidemiology, risk-benefit and cost analysis justify testing of donors and/or recipients, it is important to note that recipient follow up needs to be extended and should not be shorter than 12 weeks, as late RNA detection in non-liver recipients does occur. Conversely, in liver recipients, with the graft being the main site of virus replication, viremia becomes detectable within days from transplant.

### Understanding the Course of Infection Acquired via the Transplanted Graft

Guidance on the management of HEV in solid organ transplantation [1, 10], advise to monitor for 3 months from the point of diagnosis, unless otherwise clinically indicated, allowing time to assess the infection status and possible control without anti-viral treatment. Previous studies have indicated that approximately 33% of acutely infected solid organ transplant recipients clear HEV infection spontaneously within this time frame, with the remaining progressing to chronicity [9, 11]. Of note, subjects in the studied cohort had had their transplants years before acquiring HEV infection, a scenario that differs from when infection is acquired at the time of transplant, as the net state of immunosuppression and other parameters may differ between these time points. Whether a similar proportion of solid organ transplant patients undergoing acute donor-derived infection would have the same outcome, is unknown. In the UK cohort, only one out of the 15 individuals who tested positive for HEV RNA in plasma went on to become negative within 3 months from diagnosis of acute hepatitis. None of the patients who were either monitored beyond 3 months from the date of first positive result or who had a delayed diagnosis of HEV infection made beyond the first 3 months from transplantation, managed to control the infection and went on to receive ribavirin. This suggests that in the setting of donor-derived infection, and in contrast to infection acquired later in the post-transplant period, earlier treatment may be an approach that deserves consideration; further accrual of data from more cases may help clarifying this. Logically, this gap in knowledge and practice stems from the fact that risk of exposure through the transplanted graft can only be considered where donor screening is in place; given the variable incidence of HEV genotype 3 infection, this is a practice limited to certain regions where the epidemiology justifies such an approach. This puts countries where screening takes place, in an obligatory position to monitor the impact of the chosen strategy, follow up outcomes and use the data to inform policy and guidance.

## Conclusion

The first 5 years of universal HEV RNA screening of deceased organ donors in the UK has revealed that just under 1 in every 1,000 potential donors have confirmed HEV RNA detected in plasma due to early acute HEV infection. Donor testing and recipient follow up beyond 12 weeks has led to identification of twenty transplant recipients who were at risk of infection from the organs they had received. The majority of recipients became infected and inability to clear the virus within 3 months from diagnosis of infection was the predominant trend, except when there was intervention at an earlier point. Identification of potential exposure to the virus allowed monitoring, diagnosis and treatment, which led to control of infection in those who have completed follow up. The route and point of exposure to the virus, together with the infection dynamics in donor and recipients are known; analysis of available parameters is underway, and this will help informing the course of infection acquired via solid organ grafts, leading to a clearer understanding on how best to manage donor-derived infection in solid organ transplant recipients.

Since its inception, donor screening and recipient surveillance has ensured no donor-derived infections were missed and has allowed treatment of infections that had or would likely have evolved to chronicity. In the current UK setting, the observed yield of this screening strategy and positive impact on the outcome of organ transplant recipients indicate that the program is justified.

## Data Availability

The original contributions presented in the study are included in the article/supplementary material, further inquiries can be directed to the corresponding author.
